# Demographic and Clinical Characteristics of Patients With Charcot Neuro-Osteoarthropathy in People With Diabetes Mellitus in Six Countries: A Multicenter Observational Study From 1996 to 2022

**DOI:** 10.1155/jdr/4275741

**Published:** 2025-01-08

**Authors:** E. B. Jude, C. Siafarikas, A. Rastogi, R. Bem, T. Tankova, M.-F. Kong, J. LaFontaine, J. Pappachan, N. Tentolouris

**Affiliations:** ^1^Department of Diabetes and Endocrinology, Tameside and Glossop Integrated Care NHS Foundation Trust, Ashton Under Lyne and University of Manchester and Manchester Metropolitan University, Manchester, UK; ^2^First Department of Propaedeutic Internal Medicine, Medical School, National and Kapodistrian University of Athens, Laiko General Hospital, Athens, Greece; ^3^Diabetes and Endocrinology, Post Graduate Institute of Medical Education and Research, Chandigarh, India; ^4^Diabetes Centre, Institute for Clinical and Experimental Medicine, Prague, Czech Republic; ^5^Department of Endocrinology, Medical University of Sofia, Sofia, Bulgaria; ^6^Diabetes and Endocrinology, University Hospitals of Leicester NHS Trust, Leicester, UK; ^7^UTRGV School of Podiatric Medicine, Department of Podiatric Medicine, Surgery and Biomechanics, Harlingen, Texas, USA; ^8^Diabetes and Endocrinology, Lancashire Teaching Hospitals NHS Foundation Trust, Chorley, UK

**Keywords:** arthropathy neurogenic, Charcot joint, diabetes mellitus Type 1, diabetes mellitus Type 2

## Abstract

**Aim:** To describe the demographic and clinical characteristics of patients with Charcot neuro-osteoarthropathy (CNO) and to examine for differences between participants with Type 1 diabetes mellitus (DM) (T1DM) and Type 2 diabetes mellitus (T2DM).

**Materials and Methods:** Multicenter observational study in eight diabetic foot clinics in six countries between January 1, 1996, and December 31, 2022. Demographic, clinical, and laboratory parameters were obtained from the medical records. Analyses were performed using parametric or nonparametric statistical tests for variables with normally or nonnormally distributed values, respectively. Comparisons of the qualitative data were performed using the chi-square test.

**Results:** Seven hundred seventy-four patients with DM and CNO were included. The mean age at diagnosis of CNO was 54.5 ± 11.7 years, and the median (interquartile range (IQR)) diabetes duration at diagnosis of CNO was 15 (10–22) years. Among participants, 71.8% (*n* = 546) were male and 83.2% (*n* = 634) had T2DM. Neuropathy was present in 91.7% (*n* = 688), retinopathy in 60.2% (*n* = 452), and nephropathy in 45.2% (*n* = 337). Subjects with T1DM, compared to T2DM, were diagnosed with CNO at a younger age (46.9 ± 11.0 vs. 57.9 ± 10.2 years, *p* < 0.001), had longer diabetes duration (median value (IQR): 29.0 (21.0–38.0) vs. 14.0 (8.0–20.0) years, *p* < 0.001), and had more often microvascular complications (neuropathy, 95.2% in T1DM vs. 87.4% in T2DM, *p* = 0.006; retinopathy, 83.3% vs. 55.4%, *p* < 0.001; and nephropathy 67.5% vs. 40.5%, *p* < 0.001).

**Conclusions:** CNO is predominant in males, occurs in long-standing DM, and is often accompanied by microvascular complications. People with T1DM, compared to those with T2DM, are affected at a younger age, have longer diabetes duration, and have more often microvascular complications.

**Summary**

• Charcot neuro-osteoarthropathy (CNO) is a rare inflammatory complication of peripheral neuropathy, affecting people with diabetes mellitus (DM).
• Data about the demographic and clinical characteristics of patients with CNO are limited and often come from a single center.
• This multicenter analysis, one of the largest studies in patients with CNO, provides valuable insights into the demographic and clinical characteristics of patients with CNO.
• Individuals with Type 1 diabetes mellitus (T1DM) were diagnosed with CNO at a younger age, had longer diabetes duration, and had more often microvascular complications than Type 2 diabetes mellitus (T2DM) individuals.

## 1. Introduction

CNO is a rare but severe inflammatory complication of peripheral neuropathy that progressively leads to bone deformities as well as muscle and soft tissue injury, most often in the lower extremities [1]. CNO most commonly affects people with DM although other causes of peripheral neuropathy may also predispose to the development of this devastating condition [2, 3]. The exact prevalence and incidence of Charcot foot is unknown. Real-world data from a Danish national registry, estimated its prevalence at 0.56% of the general diabetes population [4], while in England, in seven secondary health care centers during a period of 1 month, its prevalence was estimated at 0.043% [5]. Little is known about the pathophysiology of CNO; however, according to the prevailing mechanism, loss of protective sensation due to sensory neuropathy as well as altered foot structure often leads to an uncontrolled foot and/or ankle inflammation following a minor or major trigger [3]. Diabetic foot ulcer, soft tissue infections, foot osteomyelitis, minor or major injuries, and foot surgeries have all been proposed as possible triggering situations [3]. Receptor activator of NF-kappa B (RANK), its ligand (ligand of receptor activator of NF-kappa B (RANKL)), and osteoprotegerin (OPG) have also been implicated in the inflammatory process of CNO pathogenesis by regulating osteoclast activity. OPG inhibits osteoclast activity while RANKL facilitates osteoclastogenesis and osteoclast differentiation by binding to RANK [6]. The diagnosis of CNO is often challenging and requires clinicians' awareness. The hallmark of diagnosis, the characteristic “rocker bottom” appearance where the arch of the foot is collapsed, is not always present during the initial presentation. According to a recent systematic review, the mean duration of diagnostic delay was 86.9 days while approximately half of the cases were diagnosed with delay [7]. Moreover, clinical manifestations of CNO at early stages are not specific and may resemble deep vein thrombosis, cellulitis, or arthritis. To the present, there are no available data about the prevalence and incidence of active and chronic CNO. A recent study from France and Belgium showed that chronic CNO was more prevalent than acute CNO [8]. Similar data regarding the chronicity of CNO are also available from India where chronic CNO is more frequent than acute CNO [9]. Clinical manifestations of acute CNO include redness, edema, pain, and an increase in temperature in the affected foot, while the skin is normally intact unless a diabetic foot ulcer is also present. Skin temperature, the presence of a temperature difference of at least 2°C between the affected and the contralateral foot, is frequently the initial step in making a diagnosis of CNO [1]. However, according to Raspovic et al., there is only one retrospective case series to support skin temperature difference as a diagnostic tool in CNO, and there is no evidence of the diagnostic performance of this procedure [10]. Regarding imaging, given the suspicion of Charcot's foot, plain X-rays must be performed initially followed by magnetic resonance imaging (MRI) in cases where plain X-rays are not diagnostic [1, 10].

Offloading with a knee-high nonremovable device is the mainstay of treatment of Charcot's foot to inhibit the progression of deformities and to assist dislocation and fracture healing. Removable knee-high devices, such as air casts, are an acceptable alternative option that provides the benefit of bathing and regular inspection of the affected extremity.

However, even in cases where the correct off-loading device is applied and remission is achieved, in many cases, the disease recurs. Remission is usually clinically defined as the temperature difference falling below 2^0^ C between the affected and the contralateral lower extremity. Time to remission differs significantly between different countries. A retrospective analysis in Australia that included 27 patients, reported a median resolution time of 4.3 months [11]. In the United Kingdom, Charcot's Disease in the United Kingdom study showed that median resolution time ranged between 9 and 12 months, depending on the off-loading modality [12], whilst data from the United States report a mean resolution time of 7.1 months [13]. Reactivation is defined as new-onset edema and temperature increase > 2°C between the affected and the contralateral foot [14], and its frequency has been found in different studies between 8% and 23% [15–17].

Unilateral occurrence is more prevalent, but bilateral CNO can occur as well. Armstrong et al. report bilateral involvement in 9% [13] while the EPiChar Study showed bilateral involvement was present in 22% of the patients with chronic CNO [8]. Midfoot is the most common site of CNO, accounting for 80% of total cases [8, 13–15]. Most studies use the Sanders or the Brodsky classification system, where midfoot CNO corresponds to Sanders Classes II and III [18]. To date, there are no sufficient data regarding the laterality of CNO.

The aim of this study was to describe the demographic and clinical characteristics of patients with CNO attending outpatient diabetic foot clinics. In addition, we examined for differences in CNO between people with T1DM and T2DM.

## 2. Materials and Methods

### 2.1. Diabetic Foot Clinics

We contacted 37 centers worldwide that have active outpatient diabetic foot clinics to participate in the study. Of the 37 centers, 8 centers in 6 countries responded positively and declared participation. The participating diabetic foot clinics were from the following hospitals: (1) Diabetes and Endocrinology, Tameside and Glossop Integrated Care NHS Foundation Trust, Ashton under Lyne and University of Manchester, United Kingdom; (2) Diabetes and Endocrinology, Post Graduate Institute of Medical Education and Research, Chandigarh, India; (3) First Department of Propaedeutic Internal Medicine, Medical School, National and Kapodistrian University of Athens, Laiko General Hospital, Athens, Greece; (4) Diabetes Centre, Institute for Clinical and Experimental Medicine, Prague, Czech Republic; (5) Department of Endocrinology, Medical University of Sofia, Sofia, Bulgaria; (6) Diabetes and Endocrinology, University Hospitals of Leicester NHS Trust, United Kingdom; (7) Department of Podiatric Medicine, Surgery and Biomechanics, UTRGV School of Podiatric Medicine, Texas, Texas, United States; and (8) Diabetes and Endocrinology, Lancashire Teaching Hospitals NHS Foundation Trust, Preston Road, Chorley, United Kingdom.

This was an observational study that included patients diagnosed with Charcot's arthropathy from eight Diabetic Foot Clinics in six countries between 1^st^ January 1996 and 31^st^ December 2022. The study included all patients aged > 18 years, with DM, who were diagnosed with acute CNO or followed up for chronic CNO in the outpatient diabetic foot clinics of the eight participating centers.

Since this study was observational and anonymized data were collected from the medical records, ethics approval and consent forms were not necessary.

### 2.2. Study Participants

We obtained and analyzed the data regarding demographic and somatometric characteristics such as age at diagnosis of CNO, gender, height, weight, and calculated body mass index (BMI) from the medical records during the initial visit in the reference center. We ascertained the presence and the type of DM from the medical records and we classified the participants as T1DM, T2DM, or unclassified. We also recorded diabetes duration at the time of diagnosis of CNO, based on the medical records.

### 2.3. Clinical and Laboratory Parameters

We also obtained data regarding the clinical and laboratory parameters from the medical records during the study period. We classified CNO as acute, chronic (remission), or acute on chronic (reactivation) according to clinical and imaging features based on each center's assessment. Specifically, from the clinical examination, the sudden onset of swelling, redness, and warmth of the affected foot/ankle is suggestive of acute CNO, while on the contrary, the presence of a long-standing deformity of the extremity suggests chronic CNO. In terms of imaging, X-ray radiography and MRI, where available, are commonly used to diagnose CNO. In the early stages, X-rays may be normal, and MRI is mainly used to diagnose CNO in the acute phase. In MRI bone marrow edema, joint effusion, ligament damage, and bone reabsorption with cortical fractures are indicative of acute CNO while bone sclerosis, bone deformities, and reduced bone marrow edema are suggestive of chronic CNO. Remission (active on chronic) of CNO was determined according to new edema or/and new onset elevation of temperature > 2°C in the affected extremity compared to the contralateral foot [1]. The Brodsky classification was used to classify the site of CNO as (i) midfoot (Types 1 and 2), (ii) hindfoot (Types 3A and 3B), (iii) combination of areas (Type 4); and (iv) forefoot (Type 5) [19]. We also recorded whether the affected foot was the right, left, or bilateral.

Data on diabetic microangiopathic complications such as neuropathy, nephropathy, and retinopathy, as well as laboratory data, were obtained from the medical records. For the diagnosis of peripheral neuropathy, we used the neuropathy disability score (NDS), vibration perception threshold (VPT), and the monofilament testing. Peripheral neuropathy was diagnosed when participants had an NDS ≥ 6 and/or a VPT ≥ 25 V, and/or inability to feel 5.07 monofilaments [20]. The NDS is a simple quantitative screening score used to assess diabetic polyneuropathy based on physical findings, including ankle reflex (+2), vibration sensation (+1), pinprick sensation (+1), and temperature sensation (+1), tested in each foot. A score ≥ 6 indicates peripheral neuropathy, even if symptoms are absent [20]. VPT was tested at the pulp of the hallux using a biothesiometer or a neurothesiometer, a handheld device that provides a semiquantitative assessment of large, myelinated nerve fibers [21]. A VPT value of ≥ 25 V was considered as abnormal. We calculated the estimated glomerular filtration rate (eGFR) using the Chronic Kidney Disease Epidemiology Collaboration (CKD-EPI) (2021) formula and measured the urine albumin-to-creatinine ratio (UACR) in random urine samples; we diagnosed nephropathy when the eGFR was > 90 mL/min/1.73m^2^ and UACR > 30 mg/g or when the eGFR was < 60 mL/min/1.73m^2^ regardless of UACR [22]. We reported retinopathy status based on an ophthalmological or fundus examination or if the patient had previously undergone treatment with intraocular antivascular endothelial growth factor injections or laser therapy; this information was obtained from the medical records. We reported peripheral arterial disease (PAD) when there was a history of revascularization procedures in the lower extremity arteries or when the ankle–brachial index (ABI) was < 0.9 [23]. We also obtained data on other macrovascular complications (stroke and cardiovascular disease), smoking, alcohol consumption, and other medical comorbidities from the medical records.

### 2.4. Statistical Analysis

We performed statistical analysis using International Business Machines Statistical Package for Social Sciences (IBM SPSS) Version 28.0 (IBM Corporation, Armonk, New York, United States). We checked the normality of the variables with the Kolmogorov–Smirnov test. Participants' demographic and somatometric quantitative variables with normal distribution data are presented as mean ± standard deviation, while data of variables nonnormally distributed are presented as median value with interquartile range (IQR) (25^th^–75^th^ percentile). Qualitative data are presented as *n* (percentages). For the comparison of the quantitative variables with normal distribution data between the study groups, we used the independent samples *t*-test, while for the variables with nonnormally distributed data, we used the Mann–Whitney *U* test. The comparison of the qualitative variables between the study groups was done with the chi-square test. During the processing of the data, participants with missing data in the variables of interest were excluded from the analysis for that computation (mean value, median value, or percentage). We defined statistical significance at the level of 0.05 (*p* < 0.05).

## 3. Results

### 3.1. Demographic, Somatometric, and Clinical Characteristics in the Total Sample

**Figure 1:**
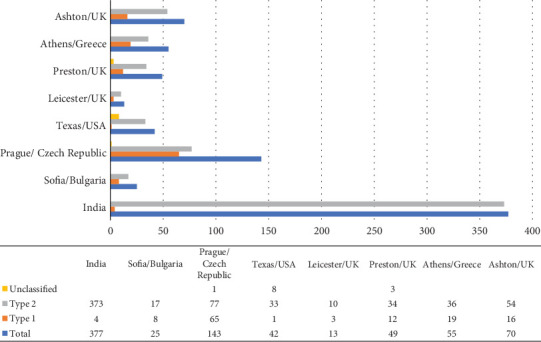
Number of study participants by country and type of diabetes mellitus.

At the end of the study, set on December 31, 2022, we included a total of 774 participants. Among the participants of this study, 128 patients were diagnosed with T1DM, and 634 patients were diagnosed with T2DM, while data about the type of diabetes were missing for 12 patients (Figure 1). Male sex and T2DM were more prevalent accounting for 71.8% (*n* = 546) and 83.2% (*n* = 634), respectively. The mean age of the patients at the time of diagnosis of CNO was 54.5 ± 11.7 years, while the median (IQR) of diabetes duration at diagnosis of CNO was 15 (10–22) years. Regarding somatometric characteristics, mean height, weight, and BMI were 169.4 ± 16.5 cm, 84.4 ± 21.0 kg, and 29078kg/m^2^, respectively. At diagnosis of CNO, the median (IQR) glycated hemoglobin was 8% (6.8%–9.3%) (Table 1).

### 3.2. Characteristics of the CNO in the Total Sample

Acute CNO was reported in 374 (50.4%) of the participants. Regarding laterality, CNO of the right foot was observed in 49.5% (*n* = 345) and of the left foot in 44%, (*n* = 307), while bilateral involvement was observed in 45 cases (6.5%). Midfoot location of CNO was observed in 65.2% (*n* = 420), forefoot in 18% (*n* = 116), hindfoot in 13.9% (*n* = 90), and multiple joint involvement in 2.6% (*n* = 18) of the subjects (Table 1).

### 3.3. Diabetes Complications and Comorbidities in the Total Sample

Concerning medical history and comorbidities, hypertension was recorded in 75.0% (*n* = 546) of the study participants, 25.2% (*n* = 184) were active smokers or ex-smokers, any alcohol use was recorded in 23.3% (*n* = 171), while PAD, coronary artery disease (CAD), and stroke were found in 19.8% (*n* = 146), 17.6% (*n* = 130), and 5.4% (*n* = 40), respectively (Table 1).

Neuropathy was the most prevalent (91.2%, *n* = 688) among the microvascular complications followed by retinopathy (60.2%, *n* = 452) and nephropathy (45.2%, *n* = 337).

The ABI was determined in 611/774 patients. No significant differences were observed between left ABI (1.013 ± 0.39) and right ABI (1.016 ± 0.40). Data regarding VPT evaluation were available for 375/774 participants. The median (IQR) VPT value was 40 V (20–50) (Table 1).

### 3.4. Comparisons Between Patients With T1DM and T2DM

Subsequently, we compared the demographic, somatometric, clinical, and laboratory characteristics of the study participants by type of diabetes (Table 1).

Patients with T1DM in comparison with those with T2DM were diagnosed with CNO at a younger age (46.9 ± 11.0 vs. 57.9 ± 10.2 years, *p* < 0.001). Moreover, at the time of diagnosis of CNO, patients with T1DM had longer diabetes duration than patients with T2DM (median value, (IQR): 29.0 (21.0–38.0) vs. 14.0 (8.0–20.0) years, *p* < 0.001) as well as lower BMI (26.6 ± 6.0 vs. 29.5 ± 8.0, *p* = 0.001). Interestingly, male sex was more prevalent in T2DM in comparison with the T1DM group (73.0%, *n* = 462 vs. 60.6%, *n* = 77, respectively, *p* = 0.04). Any alcohol consumption was more frequently reported in patients with T2DM than in T1DM (25.4%, *n* = 155 vs. 12.9%, *n* = 16, *p* = 0.003), while no significant difference was observed regarding smoking. There were no significant differences between the two groups, regarding height, weight, age at diagnosis of CNO, and glycated hemoglobin A1c (HbA1c) (Table 1).

The prevalence of diabetes microvascular complications was significantly higher in patients with T1DM. Neuropathy, the most common complication of diabetes among CNO patients was present in 95.2% (*n* = 119) of the T1DM group, compared to 87.4% (*n* = 546) in the T2DM group (*p* = 0.006). Retinopathy and nephropathy were also more frequently observed in the T1DM group (83.3%, *n* = 105 and 67.5%, *n* = 85, respectively) than in the T2DM group (55.4%, *n* = 346 and 40.5%, *n* = 251, respectively, both *p* < 0.001) (Table 1).

No significant differences were observed between the two groups regarding stroke, CAD, and hypertension. However, PAD was more prevalent in T2DM in comparison with T1DM patients (20.9%, *n* = 128 vs. 13.8%, *n* = 17, *p* = 0.04). The ABI on both right and left, although in the normal range, was significantly higher in the T1DM group than in the T2DM group (right 1.2 ± 0.23, left 1.2 ± 0.24 vs. right 0.98 ± 0.41, left 0.98 ± 0.40, feet, respectively, *p* < 0.001 for both). No significant differences were observed between the two groups regarding the VPT values (Table 1).

CNO was not different between the right and the left foot in participants with T1DM 1 and T2DM (*p* = 0.319). Regarding localization of CNO according to the Brodsky classification, midfoot was the most affected site in both groups (72.1%, *n* = 80 in T1DM vs. 63.8%, *n* = 340 in T2DM); however, in T2DM group, forefoot (19.7%, *n* = 105) and hindfoot (13.7%, *n* = 73) were the second and third most prevalent sites, respectively, while the opposite was observed in T1DM (hindfoot 15.3%, *n* = 17; forefoot 9.9%, *n* = 11) (*p* = 0.041). Acute chronic CNO was found in 15 cases (1.9%) (Table 1).

## 4. Discussion

CNO is a severe inflammatory complication of peripheral polyneuropathy, primarily affecting individuals with DM. In this study, we aimed to provide insights into the demographic and clinical characteristics of patients with CNO and to examine for differences in demographic, somatometric, and clinical characteristics between people with T1DM and T2DM.

Regarding demographic characteristics of individuals with CNO, the data from the participants in our study show that those are similar to the characteristics of patients with CNO described in the literature in similar studies [4, 8, 9, 11, 14, 15, 24–26]. According to our data, CNO is more prevalent in men than in women (70.5% men). This is consistent with previous studies conducted in various countries which also show male predominance among individuals with CNO [4, 8, 9, 11, 14, 15, 24–26].

T2DM was more prevalent in the study population (83.2%), compared to T1DM (16.8%). This finding is consistent with the epidemiology of diabetes, as T2DM is more common than T1DM [27].

The mean age at diagnosis of the CNO was 54.5 years, indicating that the condition tends to manifest in the middle-aged population. The same age distribution was noticed in an Indian retrospective study of patients with T2DM [28]. A recent population-based study from Denmark reported that the mean age of CNO patients was 60.2 years [4], while recently, a multicenter study from France and Belgium (*n* = 467 cases) and a retrospective study from Sweden (*n* = 3397 cases) showed that the mean age at diagnosis of CNO was 62 and 59.7 years, respectively [8, 25].

As expected, long-standing diabetes was observed among the study participants and the mean diabetes duration at diagnosis of CNO was 15 years. Similar duration of DM has been depicted in previous studies [8, 25, 26].

Comparing the demographic characteristics of participants with T1DM and T2DM, we found a significant (*p* = 0.004) higher prevalence of males with CNO in individuals with T2DM (73%) in comparison to those with T1DM (60.6%), a finding which is also reported in previous studies [24, 25, 29, 30].

Moreover, we found that people with T1DM tended to be diagnosed with CNO at a younger age (47.3 years in T1DM vs. 58 years in T2DM); however, this difference was not significant (*p* = 0.099). On the other hand, patients with T2DM had significantly shorter diabetes duration at the time of CNO diagnosis (14 years) compared to those with T1DM (29 years) (*p* < 0.001), a finding that is similar with previous studies [8, 25, 30] indicating that the pathogenesis of CNO may differ between T1DM and T2DM, potentially involving underlying metabolic abnormalities and comorbidities associated with T2DM or distinct mechanisms related to the autoimmune nature of T1DM. Another possible explanation for longer diabetes duration prior to CNO diagnosis in T2DM may be the fact that T2DM can remain undiagnosed for years before overt diabetes becomes prominent [31].

Somatometric measurements, such as height, weight, and BMI, provide insights into the overall characteristics of the study population. In our analysis, the mean BMI was 29.0 kg/m^2^, indicating that the participants had excess body weight. Although the association between obesity and CNO is doubtful [32, 33], several studies have shown a close, independent of hyperglycemia relationship between obesity and peripheral neuropathy [34, 35], a landmark in CNO development. However, in our study, excess body weight was more prominent in individuals with T2DM, who had higher BMI (29.5 kg/m^2^) than those with T1DM (26.6 kg/m^2^) (*p* < 0.001), indicating that overweight or obesity is a risk factor for developing CNO in T2DM. It could be hypothesized that obesity predisposes to the development of CNO due to the increased pressure on the lower extremities on one hand and, on the other hand, through to its contribution to the development of peripheral neuropathy. According to a recent study, normoglycemic individuals with obesity had lower sural nerve conduction velocity and amplitude compared to healthy normal-weight individuals and comparable peripheral neuropathy characteristics with individuals with long-standing T1DM [36].

A total of 23.3% of the study population reported alcohol consumption, which was more prevalent in T2DM patients. Previous studies have described a U-shaped association between alcohol consumption and T2DM development in men and women. [37] However, alcohol consumption has been identified as a risk factor for developing peripheral polyneuropathy due to either direct toxicity or vitamin deficiencies, such as thiamine [38]. Cases of CNO have been described [39, 40], and alcohol consumption has been associated with CNO development.

The presence of comorbidities and complications in patients with CNO is an important consideration in the management of the patients. In our study, hypertension was the most common comorbidity found in 75.0% (*n* = 546) of the participants. It has been previously noticed that individuals with diabetic foot ulcers have increased cardiovascular mortality [41]. This highlights the need for comprehensive cardiovascular risk management in patients with CNO, as hypertension is a major modifiable risk factor for cardiovascular disease.

Regarding PAD, in our study, the total prevalence of this complication in individuals with CNO was 19.8% (*n* = 146) of the total study population. Although it has been described that in CNO pathogenesis blood flow is usually increased and PAD is typically absent [3], data about the prevalence of PAD in CNO are ambiguous and, according to the literature, vary between 11% and 40% [42–44]. It is worth mentioning that data from our study show that ABI values were within the normal range in most participants, indicating that significant PAD was not a major contributing factor to the development of CNO in this population. The role of PAD in the pathogenesis of CNO remains controversial, as in the past, there were references that the presence of PAD may be protective [45]. Given that CNO is an inflammatory process, it has been hypothesized that the presence of adequate blood supply is necessary for the recruitment of inflammation cells and cytokines [45]. However, it seems that the presence of PAD (except for critical limb ischemia) cannot exclude the presence of CNO but rather adds complexity in the management of such patients, especially in the setting of a coexistent foot ulcer.

Comparing comorbidities between groups, no significant differences were observed other than PAD, which was more prevalent in T2DM. Insulin resistance, endothelial dysfunction, and inflammation, commonly implicated in T2DM pathophysiology, may partially explain the higher prevalence of PAD in this group [46].

Among the microvascular complications of diabetes, neuropathy was the most prevalent (91.7%), followed by retinopathy (60.2%) and nephropathy (45.2%). These findings underscore the close association between CNO and diabetic neuropathy, which is a major contributing factor to the development of foot deformities and subsequent complications. Notably, diabetic neuropathy was not found in all patients with CNO; this is probably due to the criteria used for the diagnosis of neuropathy which was based on examination of the large nerve fibers, and patients with small fiber neuropathy may have been missed [47]. The prevalence of all microvascular complications was significantly higher among patients with T1DM: neuropathy (95.2% in T1DM vs. 87.4% in T2DM, *p* = 0.006), retinopathy (83.3% vs. 55.4%, *p* < 0.001), and nephropathy (67.5% vs. 40.5%, *p* < 0.001), probably due to longer diabetes duration in this group.

Our data demonstrated that the midfoot is the most affected site of CNO involvement in both T1DM (72.1%) and T2DM (63.8%) groups. This distribution aligns with previous studies and is attributed to the biomechanical stress and altered foot structure in these regions [18, 48]. However, there are notable differences in the distribution patterns between the two types of DM, with the forefoot being more prevalent in T2DM and the hindfoot being more prevalent in T1DM (in T2DM forefoot 19.7%, *n* = 105 and hindfoot 13.7%, *n* = 73 vs. forefoot 9.9%, *n* = 11 and hindfoot 15.3%, *n* = 17 in T1DM, *p* = 0.041). The observed differences in the distribution patterns of CNO involvement, between T1DM and T2DM groups, suggest potential variations in the underlying mechanisms and risk factors associated with the development of this complication. Several factors may contribute to these differences, including neuropathy severity, foot biomechanics, and gait impairment between people with T1DM and T2DM. In T2DM, the higher prevalence of forefoot involvement may be attributed to the presence of excess body weight and the increased pressure on the metatarsal heads. Further research is warranted to explore these potential associations and their clinical implications.

In terms of diagnostic tools, the ABI and VPT are important parameters for assessing vascular and sensory neuropathy status, respectively. The ABI values were within the normal range in most participants. Regarding VPT, data were available for a smaller subset of participants, limiting our ability to draw definitive conclusions regarding sensory neuropathy.

This study has certain limitations. Firstly, this is an observational study, and therefore, no conclusions can be drawn regarding a causal relationship between the variables studied and CNO development. Secondly, the study population was derived from multiple centers, which could introduce variability in the assessment of CNO. Moreover, for some variables, there are missing data which can induce bias and thus downgrade the accuracy of the findings. Lastly, the generalizability of the findings may be limited to populations with similar demographic, baseline characteristics, and healthcare settings.

In conclusion, this observational study provides valuable insights into the demographic and clinical characteristics of patients with CNO and into differences between these characteristics in individuals with T1DM and T2DM. We found that CNO is more prevalent in male sex, occurs in long-standing DM, and is often accompanied by microvascular complications, while midfoot was the most affected site. Individuals with T1DM were diagnosed with CNO at a younger age and had longer diabetes duration and lower BMI than T2DM individuals. The prevalence of all microvascular complications was higher among patients with T1DM.

## Figures and Tables

**Table 1:** Demographic and clinical characteristics of the participants with Charcot neuro-osteoarthropathy.

	**Total** **n** = 774	**T1DM ** **n** = 128 **(16.8%)**	**T2DM ** **n** = 634 **(83.2%)**	**p** ** value**	**Number of subjects** **with available data**
Age (years)	56.5 ± 10.7	48.4 ± 12.4	58.1 ± 9.4	< 0.001^¶^	739/774
Age at CNO diagnosis (years)	54.5 ± 11.7	47.3 ± 10.9	58.0 ± 10.1	0.099^¶^	369/774
Duration of diabetes at CNO diagnosis, median (IQR)	15 (10–22)	29 (21–38)	14 (8–20)	< 0.001^#^	725/774
Male, *n* (%)	546 (71.8)	77 (60.6)	462 (73.0)	0.004⁣^∗^	760/774
Height (cm) (SD)	169.4 ± 16.5	174 ± 12.3	168.4 ± 17.1	0.362^¶^	584/774
Weight (kg) (SD)	84.4 ± 21.0	82 ± 20.8	84.9 ± 21.1	0.912^¶^	594/774
BMI (kg/m^2^) (SD)	29.0 ± 7.1	26.6 ± 6.1	29.5 ± 8.0	< 0.001^¶^	590/774
Any alcohol use, *n* (%)	171 (23.3)	16 (12.9)	155 (25.4)	0.003⁣^∗^	733/774
Smoke (current/ex-smokers), *n* (%)	184 (25.2)	30 (25.6)	151 (24.9)	0.475⁣^∗^	723/774
ABI right (SD)	1.016 ± 0.40	1.2 ± 0.23	0.98 ± 0.41	< 0.001^¶^	613/774
ABI left (SD)	1.013 ± 0.39	1.2 ± 0.24	0.98 ± 0.40	< 0.001^¶^	611/774
VPT, median (IQR)	40 (20–50)	35 (5–50)	40 (20–50)	0.815^#^	375/774
Retinopathy, *n* (%)	452 (60.2)	105 (83.3)	346 (55.4)	< 0.001⁣^∗^	751/774
Neuropathy, *n* (%)	688 (91.7)	119 (95.2)	546 (87.4)	0.006⁣^∗^	750/774
Nephropathy, *n* (%)	337 (45.2)	85 (67.5)	251 (40.5)	< 0.001⁣^∗^	745/774
PAD, *n* (%)	146 (19.8)	17 (13.8)	128 (20.9)	0.04⁣^∗^	736/774
CAD, *n* (%)	130 (17.6)	19 (15.3)	110 (17.9)	0.288⁣^∗^	737/774
Stroke, *n* (%)	40 (5.4)	10 (7.9)	28 (4.6)	0.097⁣^∗^	736/774
Hypertension, *n* (%)	546 (75.0)	93 (74.4)	448 (74.3)	0.540⁣^∗^	728/774
HbA1c, % median (IQR)	8 (6.8–9.3)	8.1 (6.4–9.7)	8 (6.8–8)	0.466^#^	727/774
Fasting plasma glucose (mg/dL)	126.6 ± 76.5	145.5 ± 122.8	126.4 ± 76.1	0.348^¶^	376/774
Acute CNO, *n* (%)	374 (50.4)	77 (62.1)	294 (47.6)	0.001⁣^∗^	742/774
Site, *n* (%)					
• Right	345 (49.5)	62 (50)	283 (49.4)		
• Left	307 (44.0)	50 (40.3)	257 (44.8)	0.319⁣^∗^	697/774
• Bilateral	45 (6.5)	12 (9.7)	33 (5.8)		
Specification					
• Forefoot	116 (18.0)	11 (9.9)	105 (19.7)		
• Midfoot	420 (65.2)	80 (72.1)	340 (63.8)		
• Hindfoot	90 (13.9)	17 (15.3)	73 (13.7)	0.041⁣^∗^	644/774
• Multiple joints	18 (2.9)	3 (2.7)	15 (2.8)		

*Note:* Data are shown as mean ± SD, as median (IQR), or as *n* (%).

Abbreviations: ABI, ankle–brachial index; BMI, body mass index; CAD, coronary artery disease; CNO, Charcot neuro-osteoarthropathy; IQR, interquartile range; PAD, peripheral arterial disease; T1DM, Type 1 diabetes mellitus; T2DM, Type 2 diabetes mellitus; VPT, vibration perception threshold.

^¶^ *p* values for comparisons with the independent samples *t*-test.

^#^ *p* values for comparisons with the Mann–Whitney *U* test.

⁣^∗^*p* values for comparisons with the chi-squared test.

## Data Availability

Data will be made available.

## Nomenclature

CNO: Charcot neuro-osteoarthropathy
DM: diabetes mellitus
RANK: receptor activator of NF-kappa B
RANKL: ligand of receptor activator of NF-kappa B
T1DM: Type 1 diabetes mellitus
T2DM: Type 2 diabetes mellitus
NDS: neuropathy disability score
CKD: chronic kidney disease
VPT: vibration perception threshold
UACR: urine albumin-to-creatinine ratio
HbA1c: glycated hemoglobin A1c
eGFR: estimated glomerular filtration rate
PAD: peripheral arterial disease
ABI: ankle–brachial index
CAD: coronary artery disease
